# Predicting Word Reading Deficits Using an App-Based Screening Tool at School Entry

**DOI:** 10.3389/fped.2022.863477

**Published:** 2022-05-11

**Authors:** Martin Schöfl, Gabriele Steinmair, Daniel Holzinger, Christoph Weber

**Affiliations:** ^1^Department of Educational Sciences, University of Education Upper Austria, Linz, Austria; ^2^Research Institute for Developmental Medicine, Johannes Kepler University Linz, Linz, Austria; ^3^Institute of Neurology of Senses and Language, Hospital of St. John of God, Linz, Austria

**Keywords:** word reading, primary school, predictive power, language, app-based screening

## Abstract

**Background:**

Reading is a crucial competence associated with academic development, mental health, and social adaptation. Reading difficulties are often detected at a late stage, with a possible negative impact on long-term reading development and secondary developmental disadvantages. The first manifestations of reading difficulties can be identified by word reading deficits in first and second grade, paving the way for specific interventions. For widespread implementation, instruments must be easy to use and motivating for children.

**Objectives:**

Development and validation of an economical, well-accepted, and accurate screening tool composed of the domains of phonological information processing, language skills, and non-verbal intelligence in regular school settings.

**Design:**

In 2020, the screening tool was used on a sample of 409 first graders between the second and fifth weeks of school in a one-to-one setting. Additionally, information on parental education and the use of German and/or other languages by the child was collected using a parental questionnaire. A follow-up involving the use of established standardized word reading tests was conducted at the end of the first school year.

**Results:**

A five-variable screening tool consisting of the dimensions of phonological information processing (letter knowledge, rapid naming, and phonological awareness) and linguistic skills (receptive vocabulary and morphosyntax) showed statistical relevance (AUC = 0.78; sensitivity 0.80, specificity 0.74) for predicting word reading problems concerning reading speed (<16th percentile) at the end of first grade, whereas gender, first language, and age of first exposure to the German language did not contribute to the prediction. The instrument was well accepted by the children and screeners and can be administered within an acceptable time frame.

**Conclusion:**

Word reading deficits at the end of first grade can be predicted by the use of an app-based screening tool at school entry that includes phonological information processing and language skills. Further validation and assessment of empirical feasibility data are needed to support the screening instrument for German orthography.

## Introduction

Word reading consists of several components: phonological analysis of the written word (1); orthographic processing in the sense of the “ability to form, store, and access orthographic representations” [(2), p. 4049], and lexical access to word meaning. In German orthography, which is characterized by low complexity and high transparency, reading difficulties manifest at an early stage as reduced word reading speed (3–10).

In an established reading model by Perfetti (11) and Perfetti and Hart (12), fluent word decoding and fast and effortless access to the orthographic lexicon predicted sentence and text reading fluency and thus represents an essential component of academic learning (13).

Reading weaknesses are associated with significant disadvantages throughout the school years and beyond, with impacts on school achievement (14, 15) and later employment (16). Nordström et al. (17) stressed the importance of schools investigating children’s early word decoding ability. Another large-scale longitudinal study (18) followed up individuals who had weak word decoding at the age of 7, finding that they had lower school achievement and income as adults compared to good and average readers. Stanovich summarized the logic of this finding as early as 1986 as the “Matthew Effect”: Good readers are intrinsically motivated to read and therefore read a lot, consequently, their reading skills continuously improve. Children starting school with poor reading skills often lack the motivation to read and consequently read less. Soon, a gap begins to open that is increasingly difficult to close.

Longitudinal studies have shown this very development in different orthographies [for German: (4, 5, 19, 20)].

An Australian research team conducted a comprehensive review of over 100 articles investigating the emotional consequences of slow reading in children over a period of 30 years, finding an increasingly negative impact on self-esteem and anxiety (21). German scientists replicated the findings of increased internalization of problems and resulting social withdrawal in children with reading or spelling deficits compared to children without learning disorders (22). Mammarella et al. (23) found that sustained academic failure and perceived low self-esteem increased the risk of anxiety and depression in children with reading problems. Earlier detection of risk factors connected with specific interventions could counteract this trend of reading deficits with consequences for education, employment, and wellbeing (24).

Screening tools [c.f. (25)] need to meet the following criteria: to be stable over time; to accurately predict reading achievement (criterion: “validity”); and to be objectively applicable, evaluable, and interpretable (criterion: “objectivity”). Additional criteria relate to their application in schools: screening must require little training of the test instructors; their administration should be time-efficient and administrable with limited staff resources (criterion: “test economy”). Furthermore, screening should be motivating and not overburdening for the children (criterion: “reasonableness”), and no child should be disadvantaged by the way it is conducted or the language used (criterion: “fairness”). Finally, the results should be available to teachers quickly and unambiguously and should allow conclusions to be drawn for schools, such as assignments to support groups or the adaptation of teaching methods (criterion: “usefulness”).

The use of app-based screening technology by children around school-entry age appears promising in terms of both test economy and feasibility, as demonstrated by the assessment of vocabulary performance in the last year of kindergarten (26) in Austria. Internationally, acceptable clinical screening accuracy is reported only close to or at school entry and not in the prior years (27). This is because data on the highly relevant and directly literacy-related factors (e.g., letter knowledge) can only be collected close to the beginning of school entry, in addition to more general predictors, such as non-verbal intelligence or linguistic skills.

### Child-Related Predictors

Linguistically based skills on the one hand and visual skills on the other hand have been found to predict word reading [for an overview, see (28)]. Predictors associated with visual processing such as visual memory span at kindergarten age (29) have been researched experimentally, but to our knowledge, there is still a lack of established test paradigms shown to be feasible within school-based screenings. Related to linguistically based predictors in alphabetic languages, letter knowledge, phonological awareness, and Rapid Automatized Naming-speed (RAN) have been demonstrated as robust predictors of word reading even across different orthographies and a number of reliable and practicable test paradigms have been developed (30). These factors, often summarized as phonological information processing (31, 32), are frequently supplemented by phonological working memory (33). Only recent studies have focused on the prerequisites for these factors, namely, linguistic skills. In a longitudinal study, Snowling et al. (34) demonstrated the influence of lower levels of linguistic competencies on the development of specific learning disorders. Moreover, linguistic deficits in already-diagnosed reading problems are observed retrospectively by parents more often than in average or good readers (35). Thus, asking parents about their children’s language performance and assessing it as an additional factor at school entry is anticipated to be a central component of a valid screening tool for written language skills. Non-verbal IQ as a general predictor of school success contributed little to direct variance explanation of word reading or writing performance in previous longitudinal studies. Rather, non-verbal IQ determined the level of profiles in profile analyses, such as the large-scale study by Ozernov-Palchik et al. (36). Non-verbal IQ as a general predictor of school success contributed little to direct variance explanation of word reading or writing performance in previous longitudinal studies. Rather, non-verbal IQ determined the level of profiles in profile analyses, such as the large-scale study by Ozernov-Palchik et al. (36). Latent profile analysis in kindergarteners showed specific effects and interactions of the known predictors RAN, phonological awareness, verbal working memory, and letter knowledge. The level of (non-verbal) IQ helped to identify groups of children with average or overall (non-specific) slightly below-average performance. With regard to screening IQ as an additional criterion does not lead to better identification of children that require specific reading promotion and is therefore not included.

#### Environmental Factors

Environmental factors influencing children’s reading competence have been highlighted in established reading socialization models [e.g., the multilevel model of family reading Hurrelmann et al. (37)]. They describe learners’ reading experiences in different social contexts and their influence on the development of motivation, interests, and skills. Rosebrock and Nix’s [(38), p. 16] reading literacy model includes three levels of reading competence: subjective, cognitive, and social (39). At the social level, the influence of the family as language and reading role models is emphasized as a moderator variable.

Lack of familiarity with the language spoken at school might be another factor that could affect word reading due to underspecified phonological representations or irregular letter-sound correspondence for L2 (40). Growing up with a primary language (L1) other than that used at school (L2) is usually related to having an immigrant background, including culture-specific home environments related to literacy. Although the majority of studies have found similar word reading skills in these children compared to their native peers (41–43), only a few reported better outcomes for native students (44, 45). For Dutch, there is a body of evidence in support of word decoding from kindergarten being highly comparable in Dutch as the first language (L1) and Dutch as the second language (L2) learners (46). Nevertheless, group differences at different stages of reading development have been documented, such as differences between L1 and L2 learners on rapid naming assessments in Grade 1, which disappeared in Grade 2 (47). For German (48), German L2 learners’ reading fluency was mostly predicted by non-verbal intelligence, whereas for L1 learners, phonological awareness tasks in the last year of kindergarten best predicted reading fluency.

### Scientific Aim

The aim was to construct and validate a time-efficient screening tool for word reading ability for use in community school settings around school entry. Child-related predictors concerned phonological information processing (phonological awareness, letter knowledge, and phonological working memory), language (vocabulary and grammar), and non-verbal intelligence. Children’s gender, additional environmental predictors, first language, and exposure to the German language were analyzed as potential moderating variables.

## Materials and Methods

### Participant Recruitment

The majority of the recruited children were from an Upper Austrian district with four big community-based schools. They were informed and invited to participate in the study project firstly via telephone and then by personal visits. All headmasters agreed to participate. Additionally, four more schools asked for participation and joined the project. Finally, parents of 409 children (100%) gave their written permission for including their children in the study. The final study sample reflected a heterogeneous distribution of children, comparable with Austrian primary schools in terms of gender, the proportion of children with a non-German dominant language, and parental educational levels.

**FIGURE 1:**
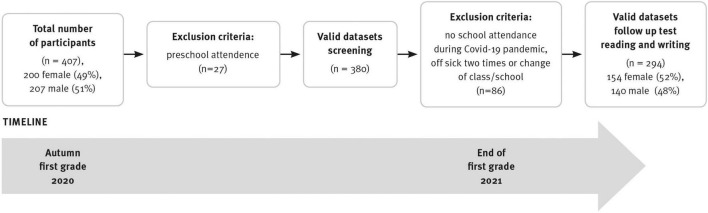
Recruitment pathways and timeline.

The individualized screening started in autumn 2020 within 2 weeks after school onset. Within 3 weeks, 85% of the sample had been assessed. In the two subsequent weeks, those children who had been ill or unable to attend during the first survey period were surveyed. A total of 27 children were not included in the analysis because they were listed as “Vorschüler” (preschoolers) in their first year of learning. A total of 86 children were sick or out of school when the reading test was conducted at the end of the first school year; these children did not differ significantly from the analyzed sample in terms of age, gender, and most importantly, the screening variables. At the end of first grade, word reading was assessed in the classroom setting. Figure 1 shows the recruitment pathways and timeline.

#### Participant Characteristics

In school statistics for the 2019/2020 school year, of the 344,282 Austrian elementary school children, 48.2% were female (49). Nearly the same proportion is found in the present study, where 48.9% (*N* = 407) are female.

In the 2019/2020 school year, 106,498 out of a total of 344,282 children in Austrian elementary schools had German not as their first language, which corresponds to 26.8% (49). Hence, around seven out of ten children in Austrian elementary schools have German as their first language [(49), p. 12]. In all nine schools studied in the project, 74.2% of children speak German exclusively as a first language. The proportions from the research project thus correspond to the Austrian distribution.

Socioeconomic status is approximated by parents’ highest educational attainment. The sample consisted of parents from all educational backgrounds: among the mothers, 4.9% had maximum educational attainment of an elementary school diploma, 14.4% had a high school diploma, and 29.8% had a university diploma. Educational levels for fathers were comparable (6.9% with maximum educational attainment of a primary school diploma, 15.4% with a high school diploma, and 21.5% with a university degree). Overall, the educational level of the Austrian population are comparable: 6% of parents have the highest educational attainment in elementary school, 22% in high school, and 27% have a university diploma. The given sample contains a variety of educational levels.

### Measures and Procedure

This 1-year prospective study followed children from the beginning to the end of first grade. The research design included two steps:

(1) Screening of phonological information processing, intelligence, and language in the first weeks of first grade, before the formal teaching of reading and spelling, had begun. The classification of the first spoken language was done by means of a questionnaire by the parents. If it was indicated that the first language was only German or contact with German occurred from birth up to and including the age of 2, then children were classified as L1. Children whose contact with German occurred only after the age of 2 were classified as L2.
(2) Standardized assessment of word reading at the end of first grade.

#### Screening Measures

The screening tool consisted of 13 subtests, which can be systematized into the three domains of phonological information processing, language, and non-verbal intelligence. A total of seven of the subtests are well-established standardized tests, whereas six of the screening tests have been newly designed; see Table 1 for an overview of the tasks. All tasks were app-supported, although for some subtests the child had a paper version to look at or an audio presentation of the stimuli was played to them on a tablet, which was used to enter results.

**TABLE 1:** Subtests and domains of the screening measures.

Domain	Subtest	Type of subtest	Number of practice items	Number of test items	Presentation mode	Target selection mode
Phonological information processing	Phonological awareness	Newly designed	3	10	Tablet	Children using tablet
	Rhyme detection					
	Phonological awareness syllable count	Newly designed	3	10	Tablet	Children using tablet
	Phonological awareness	Newly designed	3	10	Tablet	Children using tablet
	Initial phoneme detection					
	Rapid Automatized Naming, RAN (1)	Newly designed	5	30	Paper	Instructor
	Objects					
	Rapid Automatized Naming, RAN (2)	Denckla and Rudel (53) following Landerl et al. (54)	5	30	Paper	Instructor
	Digits					
	Letter knowledge	Newly designed	None	26	Paper	Instructor
	Phonological working memory	Newly designed	None	Adaptive	Tablet	Instructor
	Word list memory					
	Phonological working memory	IDS-II, Grob and Hagmann-von Arx (55)	None	Adaptive	Instructor	Instructor
	Letter–number-span forward					
	Phonological working memory	IDS-II, Grob and Hagmann-von Arx (55)	None	Adaptive	Instructor	Instructor
	Letter–number-span backward					
Linguistic skills	Receptive vocabulary	GraWo; Seifert et al. (58)	2	30	Tablet	Children using tablet
	Sentence repetition	Adapted from Hamann and Abend Ibrahim (57)	None	15	Tablet	Instructor
Intelligence	Complete matrices	PITVA (59)	None	Adaptive	Paper	Instructor
	Picture series	PITVA (59)	None	Adaptive	Paper	Instructor

##### Phonological Information Processing

Phonological awareness was assessed by three tasks, with one task intended to differentiate in the lowest performance range (rhyming), one in the middle (syllable count), and one close to written language acquisition (initial phoneme detection). Phonological awareness tasks were newly constructed despite the presence of existing tests in order to meet the quality criterion of the economy for the use of the instrument in the school setting. Existing test procedures in German-speaking countries are well constructed but are too time-consuming for universal use in schools [e.g., (50)]. Each of the tasks was introduced by three practice items including feedback, followed by ten test items. Tasks were constructed from high-frequency words from the childLex database (51) for the youngest age group (6–8 years). Syllable count was controlled for the target items, and distractor tasks consisted of phonologically similar structures to the target items.

For the rhyming task, the child selected the words that rhyme from a set of three words (picture and word presented). Ten examples (7 one-syllable and 3 two-syllable) were presented. For example: “What rhymes: house, mouse, man?” An explorative factor analysis (EFA) for binary items conducted with Mplus 8 (52) showed a dominant factor with an eigenvalue of 5.351 (53% explained variance). A second factor with an eigenvalue of 1.418 was not interpretable. Moreover, the one-factor solution yielded an acceptable fit [χ^2^(35) = 0.9405, root mean square error of approximation (RMSEA) = 0.065; comparative fit index (CFI) = 0.918]. Therefore, we choose the single-factor solution. Internal consistency was (Kuder–Richardson KR-20) low but acceptable at 0.610. Internal consistency was (Kuder–Richardson KR-20) low but acceptable at 0.61.

Syllable count was also assessed by one- to four-syllable words, presented by means of pictures and spoken language; visual cues (one clapping a hand to four clapping hands) were used to indicate the number of syllables. Notably, an EFA yielded an inadequate fit for a one-factor solution [χ^2^(35) = 211.4, RMSEA = 0.112, CFI = 0.850], but a good fit for a two-factor solution [χ^2^(26) = 38.9, RMSEA = 0.035, CFI = 0.989]. The analyses revealed a factor focusing on two or more syllable words (7 items; eigenvalue = 4.563, 46% explained variance) and a factor focusing on one-syllable words (3 items; eigenvalue = 1.876, 19% explained variance). Thus, we used two different syllable count scores in this paper. Internal consistency was good for the one-syllable factor (KR-20 = 0.764) and acceptable for the two or more-syllable factor (KR-20 = 0.651).

For initial phoneme detection, we presented a letter visually and as a speech sound simultaneously.

From a selection of three pictures, those with the same first phoneme had to be selected (“Which word begins with I like Ines: Hase, Igel, Spiegel?”). Although an EFA yielded three factors with eigenvalues greater than 1 (3.772, 1.418, 1.064), the one-factor solution showed an adequate fit [χ^2^(35) = 74.3, RMSEA = 0.053, CFI = 0.908]. Thus, we choose the single-factor solution. Internal consistency was adequate (KR-20 = 0.632).

For the assessment of Rapid Automatized Naming (RAN), two conditions were chosen: objects and digits. The RAN object condition was designed through five high-frequency monosyllabic words (cow, hand, ice, tree, and mouse). First, the items of the RAN tasks were presented app-based, and the task was given to repeat these items. Once the investigator ensured that the instruction was understood and the items were known, the test session started. The items were presented on paper repeatedly in a different order over six lines. The investigator pressed a button on the tablet to time the test and noted any incorrect responses on the tablet by pressing a button. When the last item was reached, the time measurement was stopped manually again, and the time distance was calculated automatically.

Rapid Automatized Naming in the digit condition was based on the work of Denckla and Rudel (53), following (54), and was presented and rated analogously to the object condition with monosyllabic digits (2, 8, 1, 6, 3).

Letter knowledge: All letters of the alphabet were offered as capital letters in random order on paper. Each page contained three to four letters. Children were asked: “I know you haven’t learned these letters yet at school. Maybe you still know one? Please name it!.” Positive scores were given for letter names or sounds and ticked off on the tablet.

Phonological working memory was assessed by two subtests of a broad-range intelligence test battery [IDS-2; Intelligence and Development Scales for Children and Adolescents, (55)] testing memory of letter-number sequences forward and backward. The child was asked to repeat a series of digits mixed up with letters (3-A, 5-M-2) in the same (forward condition) way or form back and forth (backward condition). The investigator clicked correct solutions on the tablet. The difficulty level of the tasks was determined by the length of the spans, and the termination occurred after three unsolved or incorrectly solved tasks. The longest possible range of letter and number sequences was of the target value. Reliability is described as fair; Cronbach’s Alpha was 0.89 (end of first grade). Retest reliability was rtt = 0.93 (first grade).

Wordlist memory was also used to analyze phonological working memory: a list of 10 words (5 single-syllable words and 5 two-syllable words) was presented *via* an audio file. The child was then asked to freely reproduce as many of them as possible. The investigator ticked off the words in the mentioned order (including repetitions and wrong words). The sum of all memorized items yielded the overall performance.

##### Language

Morphosyntactic skills were assessed by an adapted sentence-repetition task. The German version was constructed according to the LITMUS (Language Impairment Testing in Multilingual Children) principles (56) by Hamann et al. (57) following the COST Action IS0804. A block of 15 items representing morphosyntactic constructions with varying degrees of complexity was selected and scored according to whether or not the sentence was completed correctly. Correctness was judged and noted in the app by the examiner when the sentence structure was reproduced completely correctly, regardless of articulatory deficits.

Internal consistency (KR-20) was high at 0.877.

In a digital form of the Graz Vocabulary Test [GraWo; (58)], receptive vocabulary was tested by 30 matching tasks. The child was required to choose from four pictures the one that matched the audio-presented word. Reliability data are given for the paper form of the GraWo: Cronbach’s Alpha ranged from 0.89 (end of first grade) to 0.82 (end of second grade). Retest reliability was rtt = 0.93 (first grade).

##### Non-verbal Intelligence

Two subtests of the PITVA (59) were used to assess non-verbal intelligence: Complete Matrices (Cronbach’s Alpha 0.83 for 6 year old/Retest reliability rtt = 0.9) and Picture Series (Cronbach’s Alpha 0.86). The child was shown matrices and sequences of items and had to click on the correct condition from a selection directly on the tablet.

#### Word Reading

Word reading and writing tasks were administered in a classroom setting at the end of the first school year, exclusively by research staff.

The ELFE II word reading test (60) was used to assess decoding fluency at the word level in silent reading. For each picture, the appropriate written word from a selection of four had to be selected. The test duration was limited to 3 min. Representative norm scores are available from the end of the first school year to the beginning of the seventh grade; reliability data are presented as excellent (split-half *r* = 0.98, retest *r* = 0.83). A cut-off score of 13 represents M – 1 SD.

### Procedure

Before the implementation of the screening tool, the principals of participating schools received information about the testing process. They were also given a letter to send to parents, including consent forms and questions about children’s first language and language use as well as parents’ educational background.

Teachers entered children’s names into an online database, which converted the names to IDs for use on the tablet. The testers, all of them were student teachers, were enrolled in a student seminar in order to learn about the tasks and testing procedure, through which the teachers received student credit for the study (amounting to 4 h). The materials for testing were brought to the schools by a research coordinator. On the test mornings, it was agreed with the school administration that the children would be selected alphabetically by the test team (student teachers and core study project staff) from the classrooms. The assessment took place one at a time, with the child and instructor seated across a table from each other. After a brief welcome, the child was handed the tablet, in which the friendly dragon SCHWUPP was introduced right at the beginning. The app navigation was designed in a way that the child can use it independently, but if necessary, the test leader intervened in the navigation of the dragon from one task to the next. All instructions essential for the child were recorded as audio files, opened automatically, and could be repeated if necessary. A yellow background on the app signaled to the test administrator that the child was making test selections independently (such as in the phonological tasks). A gray background meant that the test administrator had to take the tablet to read the instructions from the tablet and give the corresponding instructions. This was especially true for tasks with material (for example, the letter cards). The assessment including all subtests took an average of 38.4 min (SD = 9.3) per child.

## Methods of Analysis

First, we used receiver operator characteristic (ROC) analyses to evaluate the diagnostic accuracy of each subscale. Following Swets (61), AUCs ≥ 0.9 are regarded as excellent, AUCs ≥ 0.8 and <0.9 as good, AUCs ≥ 0.7 and <0.8 as fair, and tests with AUCs < 0.7 as poor. ROC analyses were conducted using the pROC package (62) in R.

Second, to construct a time-efficient screening to predict word reading difficulties at the end of Grade 1, we used a logistic regression model with adaptive variable selection to identify important subtests. In detail, we applied the least absolute shrinkage and selection operator [LASSO; e.g., (63, 64); for an application of LASSO for the selection of screening variables, see (65)] as implemented in the glmnet R Package (66), which adequately addresses the problem of overfitting that is pronounced in standard variable selection procedures (e.g., backward or forward selection) and models with many predictors (relative to the sample size). Overfitting occurs when sample regression estimates capture signal and noise and thus are larger than in the population, which in turn limits the generalizability of the regression results. LASSO addresses overfitting and consequently increases generalizability by applying a penalty term (λ) to the likelihood function that protects estimates from inflation. Just as in the backward or forward selection, null predictors are zeroed out (i.e., they are excluded from the prediction model). Notably, LASSO does not provide *p*-values (methods have been developed for linear models, but not for logistic models) and thus it is not possible to refer to the “significance” of predictors. Instead, the selected predictors are meaningful whether their effects are significantly different from zero or not (63, 65).

To evaluate the importance of the screening variables, we z-scored the predictors. Thus, reported estimates are in a standardized metric. Moreover, LASSO requires the selection of an appropriate penalty term. We used 10-fold cross-validation and selected the value for λ that resulted in the highest area under the curve (AUC). Since in some cases this may insufficiently address the problem of overfitting, we also report results for the second value of λ by applying the one standard error rule [i.e., selecting the largest value of λ at which the AUC is within one standard error of the largest AUC; see e.g., (64), p. 216]. Once a set of predictors had been selected, we used the regression coefficients of the LASSO models to estimate the probability of scoring within the 10%-percentile of the word reading test at the end of Grade 1. This probability score (ranging from 0 to 1) was subsequently used as a screening measure.

Third, we compared the screening scores based on the results for λ at the maximum AUC with scores based on the one standard error rule by applying a bootstrapped test that compares the AUCs of paired ROC curves (62).

Fourth, we compared ROC curves between groups defined by the first language (German vs. non-German), German language exposure (≤2 years vs. >2 years), and gender (girls vs. boys). Significantly differing ROC curves between groups indicate variations in diagnostic accuracy, which would consequently limit the generalizability of the screening tool (67). Besides using a bootstrapped test for unpaired ROC curves that compare the AUCs for two groups, we also applied the Venkatraman permutation test (68) that compares actual ROC curves (also implemented in the pROC package). If two ROC curves do not differ significantly between groups, screening scores would yield the same sensitivity and specificity in both groups, and thus, a single cut-off for both groups would be appropriate.

Finally, we determined optimal cutoff scores using the R-OptimalCutpoints package (69). Cut-offs were evaluated based on the following diagnostic accuracy statistics: sensitivity (Se), specificity (Sp), positive predictive values (PPV), negative predictive values (NPV), and diagnostic likelihood ratios for positive and negative screening results (DLR+ and DLR−, respectively). Se and Sp ≥0.9 indicate good diagnostic accuracy, Se and Sp ≥0.80 are regarded as fair, and values below 0.80 indicate an unacceptably high rate of misclassification (70). DLR+ and DLR− are diagnostic accuracy measures that—unlike predictive values—do not depend on the prevalence of the disorder under investigation (71). DLR+ displays the multiplicative change in the pre-screening odds of scoring in the 10%-percentile of the reading test given a positive screening result (i.e., post-screening odds = DLR+ × pre-screening odds). DLR− is the change in the pre-screening odds of scoring in the 10%-percentile given a negative screening result (post-screening odds = DLR− × pre-screening odds). DLR+ values ≥10 and DLR−≤0.1 indicate large changes in pre-screening odds, DLR+ ≤10 and >5, and DLR−>0.1 and ≤0.2 indicate moderate changes, DLR+ ≤5 and >2, and DLR−>0.2 and ≤0.5 indicate small changes. DLR+ <2 and DLR−>0.5 are rarely important (72).

## Results

Table 2 (Section A) shows the AUCs as well as the point-biserial correlations (r_*pb*_) for the screening subtests. Notably, rhyme detection, syllable count, letter–number sequences forward, and the IQ subtests are not significantly associated with word reading problems at the end of Grade 1. For all other predictors, correlations are small and only the AUCs for RAN (digits and objects) and letter knowledge could be regarded as fair. Overall, the AUC for RAN objects is largest at 0.726 (DeLong 95%-CI [658, 0.795]), directly followed by letter knowledge (0.723, DeLong 95%-CI [0.645, 0.801]).

**TABLE 2:** Areas under the curves (AUCs) for subtests and results of the LASSO logistic regression models.

	Section A			Section B	
				Lasso Model 1 – AUC = MAX	Lasso Model 2 – 1 SE rule
				
	r_pb_	AUC	95%-CI DeLong	Estimate (OR)	Estimate (OR)
**Phonological awareness**					
Rhyme detection	–0.042	0.521	(0.433, 0.610)		
Syllable count (one syllable)	–0.075	0.552	(0.467, 0.637)		
Syllable count (two or more syllables)	–0.086	0.555	(0.467, 0.643)		
Initial phoneme detection	−0.211***	0.663	(0.588, 0.738)	−0.061 (0.941)	
**Rapid Automatized Naming**					
RAN objects	0.287***	0.726	(0.658, 0.795)	0.312 (1.366)	0.174 (1.190)
RAN digits	0.255***	0.717	(0.632, 0.802)	0.098 (1.103)	
**Letter knowledge**					
Letter knowledge	−0.280***	0.723	(0.645, 0.801)	−0.380 (0.684)	– 0.179 (0.836)
**Phonological working memory**					
Word list memory	−0.149**	0.601	(0.517, 0.686)		
Letter–number sequences forward	–0.084	0.589	(0.506, 0.671)		
Letter–number sequences backward	−0.184***	0.627	(0.541, 0.714)		
**Linguistic competences**					
Vocabulary	−0.184***	0.615	(0.523, 0.706)		
Sentence repetition	−0.189***	0.642	(0.556, 0.727)	−0.040 (0.961)	
**Intelligence**					
Subtest A	–0.072	0.581	(0.504, 0.658)		
Subtest B	–0.095	0.566	(0.477, 0.654)		
Intercept				–1.675	−1.567

**p < 0.01, ***p < 0.001.

The results of the LASSO logistic regression models are reported in Table 2 (Section B). When selecting the value for the penalty term λ that yields the highest AUC (Model 1), the model identifies five non-zero predictors: initial phoneme detection (*b* = −0.061), RAN objects (*b* = 0.312), RAN digits (*b* = 0.098), letter knowledge (*b* = −0.380), and sentence repetition (*b* = −0.04). Remember that all subtests were z-scored, thus the strength of the regression coefficients could be directly compared. Applying the one standard error rule for the selection of λ (Model 2) results in two non-zero predictors. RAN objects (*b* = 0.174) and letter knowledge (*b* = −0.179)—the strongest predictors of Model 1—were selected as meaningful predictors.

The screening score based on LASSO Model 1 yields an AUC of 0.783 (DeLong 95%-CI [0.713, 0.852]), and the AUC for the screening score based on LASSO Model 2 is 0.773 (DeLong 95%-CI [0.704, 0.843]). A bootstrapped test for paired ROC curves indicates that the AUC-Difference is statistically not significant (ΔAUC = 0.01, *D* = 1.317, *p* = 0.188). However, as AUC-difference tests are known to be plagued with low power [e.g., (73)], we decided to further evaluate the screening scores based on LASSO Models 1 and 2.

Table 3 reports the results for the comparison of ROC curves between groups. The tests for unpaired ROC curves show no significant differences between the groups. Thus, the screenings based on the LASSO selected predictors show no differences in diagnostic accuracy between German and non-German-speaking children, children with German-language exposure ≤2 years, children with German-language exposure >2 years, and girls and boys.

**TABLE 3:** Comparing receiver operating characteristics (ROC) curves between subsamples.

		LASSO Model 1			LASSO Model 2		
		AUC	95%-CI	AUC-Difference (2)	AUC	95%-CI	AUC-Difference (2)
First language	German (1)	0.768	(0.671, 0.866)		0.761	(0.664, 0.857)	
	Non-German (2)	0.786	(0.680, 0.892)	*E* = 0.038, *p* = 0.502 *D* = 0.248, *p* = 0.804	0.776	(0.667, 0.885)	*E* = 0.041, *p* = 0.417 *D* = 0.202, *p* = 0.8401
German language exposure	≤2 years (1)	0.767	(0.681, 0.856)		0.760	(0.674, 0.846)	
	>2 years (2)	0.810	(0.687, 0.934)	*E* = 0.015, *p* = 0.780 *D* = −0.550, *p* = 0.582	0.790	(0.659, 0.920)	*E* = 0.014, *p* = 0.808 *D* = −0.385, *p* = 0.700
Gender	Boys (1)	0.755	(0.643, 0.866)		0.744	(0.632, 0.856)	
	Girls (2)	0.809	(0.721, 0.897)	*E* = 0.017, *p* = 0.695 *D* = −0.754, *p* = 0.451	0.803	(0.716, 0.890)	*E* = 0.02, *p* = 0.576 *D* = −0.834, *p* = 0.405

*E-value for AUC-Difference refers to the Venkatraman test that compares ROC curves and the D-value refers to the bootstrapped test for paired ROC curves that compares AUCs.*

Finally, we estimated cutoffs for both screening scores by setting the sensitivity equal to 0.80. This cutoff was chosen to achieve acceptable sensitivity while holding the rate of positive screens as low as possible. The diagnostic accuracy statistics are reported in Table 4. Both screening scores yield a sensitivity of 0.808 (i.e., the cutoff value that achieves a sensitivity closest to 0.8). For Model 1, the cutoff is 0.195. This cutoff results in 36.3% of screening fails. Notably, given that the screening scores based on Model 1 and Model 2 achieve an identical sensitivity, the other diagnostic accuracy statistics are favoring Model 1. Importantly, as indicated by a significant McNemar Test [χ^2^(1) = 4.923, *p* < 0.05], the Model 1 screening turns out to be significantly more specific than the Model 2 screening (Model 1: Sp = 0.733, 95%-CI [0.672, 0.787], Model 2: Sp = 0.695, 95%-CI [0.633, 0.753]).

**TABLE 4:** Diagnostic accuracy statistics.

	Se (95%-CI)	Sp (95%-CI)	PPV (95%-CI)	NPV (95%-CI)	DLR+ (95%-CI)	DLR− (95%-CI)
LASSO Model 1	0.808 (0.675, 0.904)	0.733 (0.672, 0.787)	0.393 (0.326, 0.591)	0.947 (0.898, 0.960)	3.012 (2.360, 3.865)	0.263 (0.150, 0.461)
LASSO Model 2	0.808 (0.675, 0.904)	0.695 (0.633, 0.753)	0.362 (0.300, 0.559)	0.944 (0.893, 0.957	2.652 (2.104, 3.344)	0.277 (0.157, 0.486)

For Model 2, the cutoff of 0.186 results in 39.3% of screening fails. Given that the screening scores based on Model 1 and Model 2 achieve an identical sensitivity, the other diagnostic accuracy statistics of Model 1 are better than those of Model 2. Notably, as indicated by a significant McNemar Test [χ^2^(1) = 4.923, *p* < 0.05], the Model 1 screening turns out to be significantly more specific (Model 1: Sp = 0.733, 95%-CI [0.672, 0.787], Model 2: Sp = 0.695, 95%-CI [0.633, 0.753]).

## Discussion

### Constructing a New Screening Battery

The analysis of a broad battery of subtests aiming to predict word reading deficiencies at the end of first grade resulted in two models: a short one consisting of two subtests (AUC = 0.77) and a broader one with five subtests (AUC = 0.78). Both models include a task for rapid naming and letter knowledge; the broader version additionally includes two language subtests (vocabulary and grammar) and a short assessment of phonological awareness (first phoneme detection).

There is the general consensus about the acceptable test accuracy of developmental screenings, namely, a sensitivity of 0.80 and specificity of 0.70 (74, 75). Thus, moderate specificity values may be acceptable, but high sensitivity is demanded for universal screening (76, 77). Setting a sensitivity range of 0.80, the specificity of the short version with two predictors is 0.69, and the specificity of the extended model with three additional subtests is 0.74. Yielding significantly higher specificity, the extended model is thus the preferred model.

The positive predictive value of Model 1 is 0.36; that is, 36% of the children with low screening results are in the slow readers’ group at the end of Grade 1. The preferable five-variable model identifies 39% of the children with low reading results at the end of first grade, identifying 42 children at risk as low readers correctly. The achieved predictive values represent an improvement compared to the only recent assessment for preschoolers in German, the “LRS-Screening” (50) which uses a range of 14 subtests in the year prior to starting school to predict word reading deficits at the end of first grade, with a sensitivity of 0.74, a specificity of 0.68, and a PPV of 0.27. The “LRS-Screening” is presented in a one-to-one setting in a paper–pencil version. It lasts for a duration of about 30 min, requires additional scoring time, and does not provide a cover story that would be assumed to make the assessment more appealing. In comparison, the five-component model of the newly developed screening tool can now be administered in about 15 min, including a shortened cover story, and is the only app-based screening tool for the early identification of reading problems in German. The “Bielefelder Screening” (78) is widely used in the year prior to school entry. In the manual, good predictive values (as high as 50%) are quoted, which could not be replicated by independent studies (79). Another screening, designed for group assessment during the last year of kindergarten, is the Phonological Awareness-Reading and Spelling Screening [PB-LRS; (80)]. The authors reported a sensitivity of 63%, specificity of 87%, and PPV of 36%. The duration of this screening tool with acceptable predictive quality is about 60 min.

Another established screening tool is called “Tour through Hörhausen” (81), which provides a phonological assessment of children in one-to-one settings at the beginning and the midpoint of the first grade. Prognostic validity was analyzed using a sample of 375 children, focusing on word reading speed at first grade. The authors described its specificity as over 80%, whereas the sensitivity varies between 38 and 48%. The assessment time is about 40 min. In summary, established screenings to predict reading difficulties in German require a long administration time and demonstrate low predictive power [for an overview, see (82)].

With the five-component model, there is no significant difference in the prediction of reading deficits according to gender or first language (German or non-German). Therefore, no specific cutoffs for gender or first language are needed.

Phonological awareness is one component of phonological information processing that is highly significant for the prediction of reading deficits in the international English-dominated literature [e.g., (83) for an overview]; in more consistent orthographies, such as Italian or German, word reading deficits are primarily predicted by the measures of letter knowledge and RAN (54, 84, 85). The prediction of reading performance at word level by vocabulary and grammar, summarized as linguistic competencies (34), was confirmed in the present study for the German language.

Interestingly, the factor of non-verbal intelligence plays a subordinate role; in the statistical model, it does not attain significance. In German-language longitudinal studies, the predictive quality of non-verbal intelligence on reading fluency was minimal (86, 87). As in the present study, factors specific to reading and writing, such as RAN and letter knowledge, showed higher predictive power for reading difficulties than the general factor of non-verbal intelligence.

Family history of reading problems was not included in our analysis, although prior studies found some contribution to a prediction model (36). However, a recent longitudinal study with a representative, epidemiological sample did not report acceptable AUC values for predicting reading problems by eliciting family risk factors (88), therefore diminishing the predictive value of family risk factors. This effect is expected especially for a German-speaking country because there are usually reservations about reporting family predispositions, and therefore no or unreliable information is provided.

### School Use of the Screening Battery

For use in schools, screening tools should not only have high predictive power and reliability but also should have applicability with limited resources. In addition, screenings should be highly motivating for children. Children indicated that they experienced the assessment as a game and were able to stay with it well over a median duration of about 38 min. Not a single child had to stop for reasons of motivation or declining attention. The identification figure SCHWUPP provided continuous positive feedback after each completed subtest, and the frame story between the tasks could be used for relaxation. The new screening tool can be administered in about 15 min, making it shorter than any other screening tool available for the German language.

For the testers themselves, a high degree of objectivity was ensured because all instructions were played as audio files and important additional information (e.g., when naming the letters) was documented in the app. Due to the high degree of standardization, the training effort was low. Given that the one-to-one test setting remains necessary since some tasks require a paper target (such as letter knowledge or RAN) and the screening tool is for young children, a contact person is important in stressful situations. For teachers, rapid feedback of the results through automatic uploading of the results and further evaluations by the project team was important. Furthermore, for teachers, automatic scoring is regarded as a key feature of a feasible instrument.

### Strengths of the New Screening Tool

The comprehensive sample is representative of Austria and the German language. A five-variable screening for surveillance in a community school setting showed good predictive power to detect slow readers at the end of first grade. For children, a motivating cover story presented interactively through tablets helps to maintain their motivation through a series of tasks. Advantages for screeners are short administration time, objectivity, and a quick computation of results. In order to make the whole screening tool available to primary schools without licensing costs, sub-tests had to be newly designed. There is now a screening tool that meets these requirements.

### Limitations

Although a PPV of 39% is good compared to given screening tools, it does not cover all the children with slow reading at the end of first grade. Some data about environmental factors have been captured, but a big amount of variance is still to be detected: reading socialization *via* parents, school, friends, and at a macro level, society. With regard to the predictors, it must be noted that only language-related variables were included. Evidence on preschool visual processing has also recently been shown to be predictive of the reading process. Visual predictors were unfortunately not collected in the present study. Finally, continuous surveillance of reading is required because there might be different pathways to reading difficulties (many children with early difficulties do not develop later reading problems, and many children who do not fail the initial screening demonstrate reading difficulties later on).

### Implications for Research and Practice

The first steps for a new screening on reading deficits have been implemented. Further validation of the newly constructed screening is needed, the next steps include a bigger normative sample and comparisons with screening tools already in use. Feasibility data for school usage must be gathered from children and teachers in order to enhance the screening and support a broader and well-accepted rollout.

## Data Availability Statement

The original contributions presented in the study are included in the article/Supplementary Material, further inquiries can be directed to the corresponding author.

## Ethics Statement

The studies involving human participants were reviewed and approved by the Regional School Board for Upper Austria (Bildungsdirektion Upper Austria). Written informed consent to participate in this study was provided by the participants’ legal guardian/next of kin.

## Author Contributions

MS and GS: conceptualization, investigation, data curation, and project administration. MS, GS, and CW: methodology. CW: formal analysis. MS, GS, CW, and DH: writing original draft preparation and reviewing and editing. All authors contributed to the article and approved the submitted version.

## Conflict of Interest

The authors declare that the research was conducted in the absence of any commercial or financial relationships that could be construed as a potential conflict of interest.

## Publisher’s Note

All claims expressed in this article are solely those of the authors and do not necessarily represent those of their affiliated organizations, or those of the publisher, the editors and the reviewers. Any product that may be evaluated in this article, or claim that may be made by its manufacturer, is not guaranteed or endorsed by the publisher.
