# Supplementary material for: Predicting Word Reading Deficits Using an App-Based Screening Tool at School Entry
Source: Front Pediatr. 2022 May 11;10:863477. doi: 10.3389/fped.2022.863477 (PMC9130720; doi:10.3389/fped.2022.863477)
Supplement: Supplementary file 1 [file Data_Sheet_1.PDF]

Supplementary Material

1 Supplementary Figures

Hereinafter, we present supplementary figures on the distribution of the screening variables. Plots were created using the Survey Plots Package in Jamovi 2.2. (The jamovi project, 2021).

Figure 1: Distributions of the Screening Tests (standardized scores)

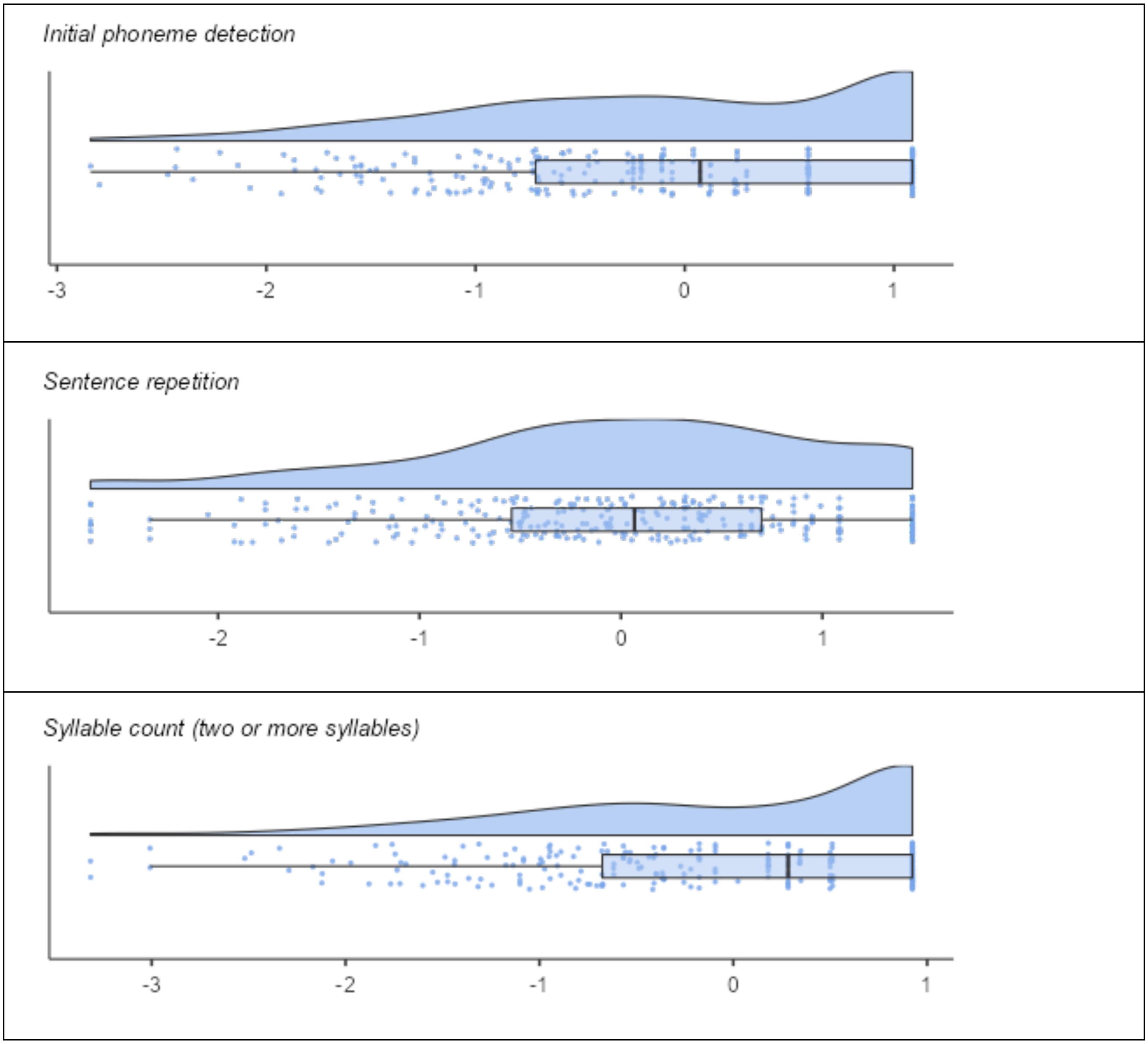

Syllable count (one syllable)

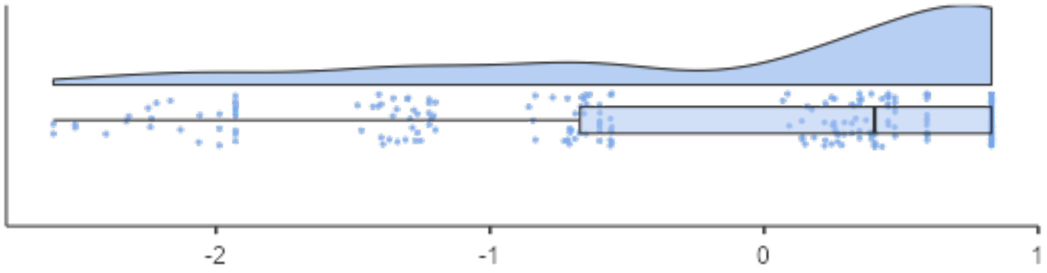

Syllable count (one syllable)

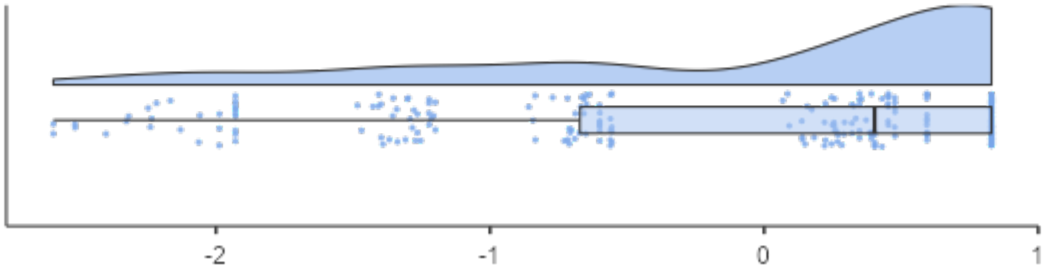

Rhyme detection

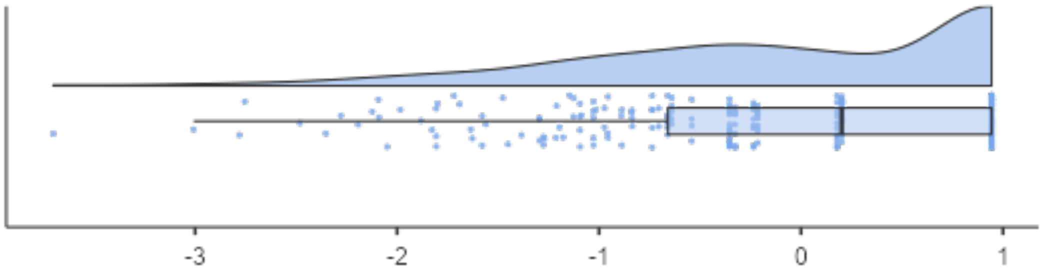

Vocabulary

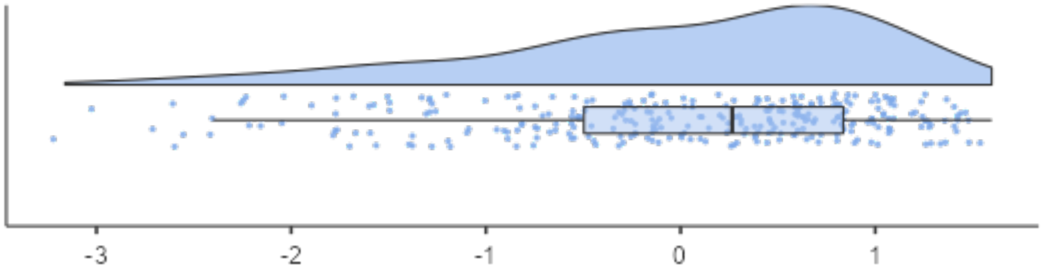

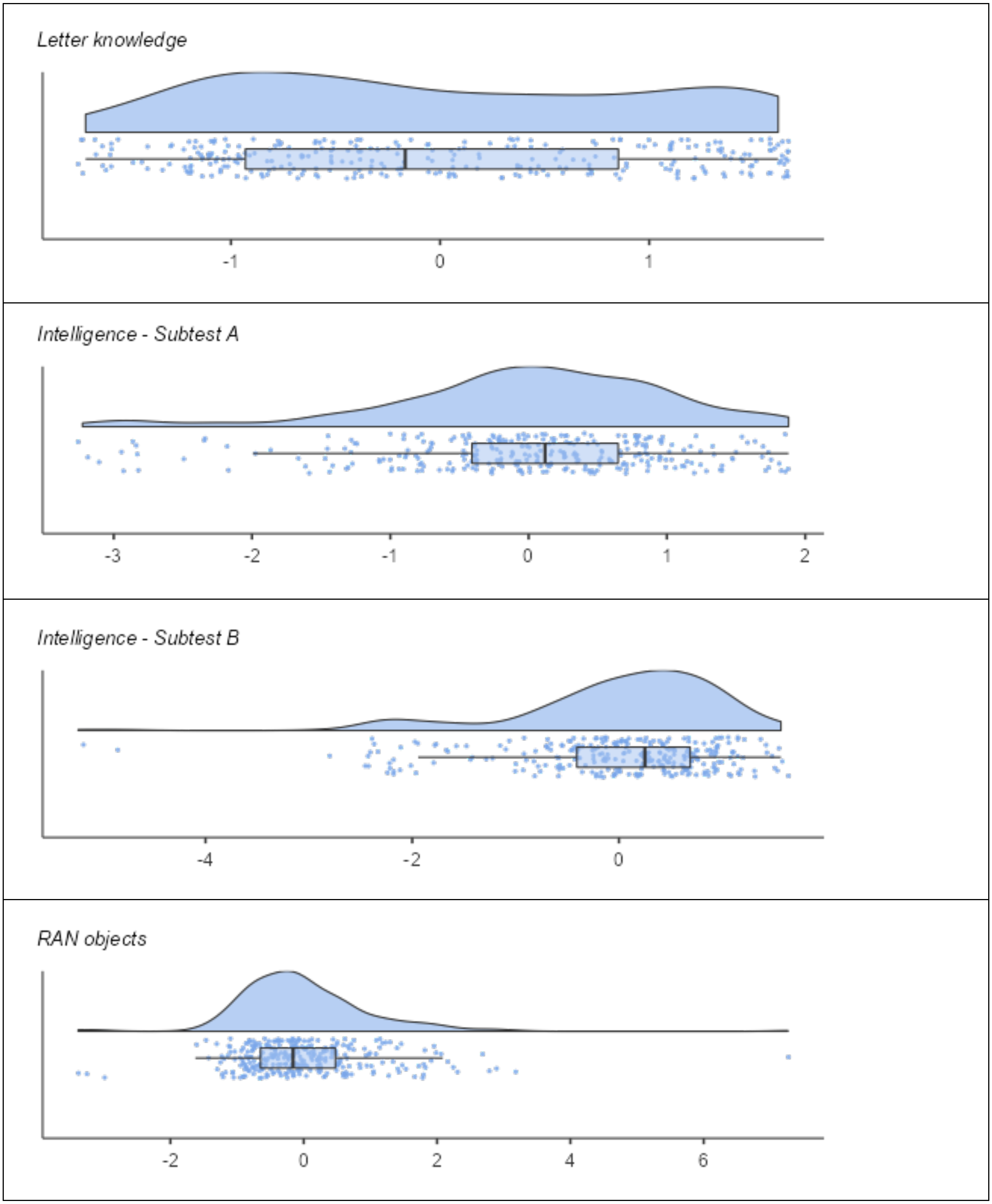
Letter knowledge
-1
0
1
Intelligence - Subtest A
-3
-2
-1
0
1
2
Intelligence - Subtest B
-4
-2
0
RAN objects
-2
0
2
4
6

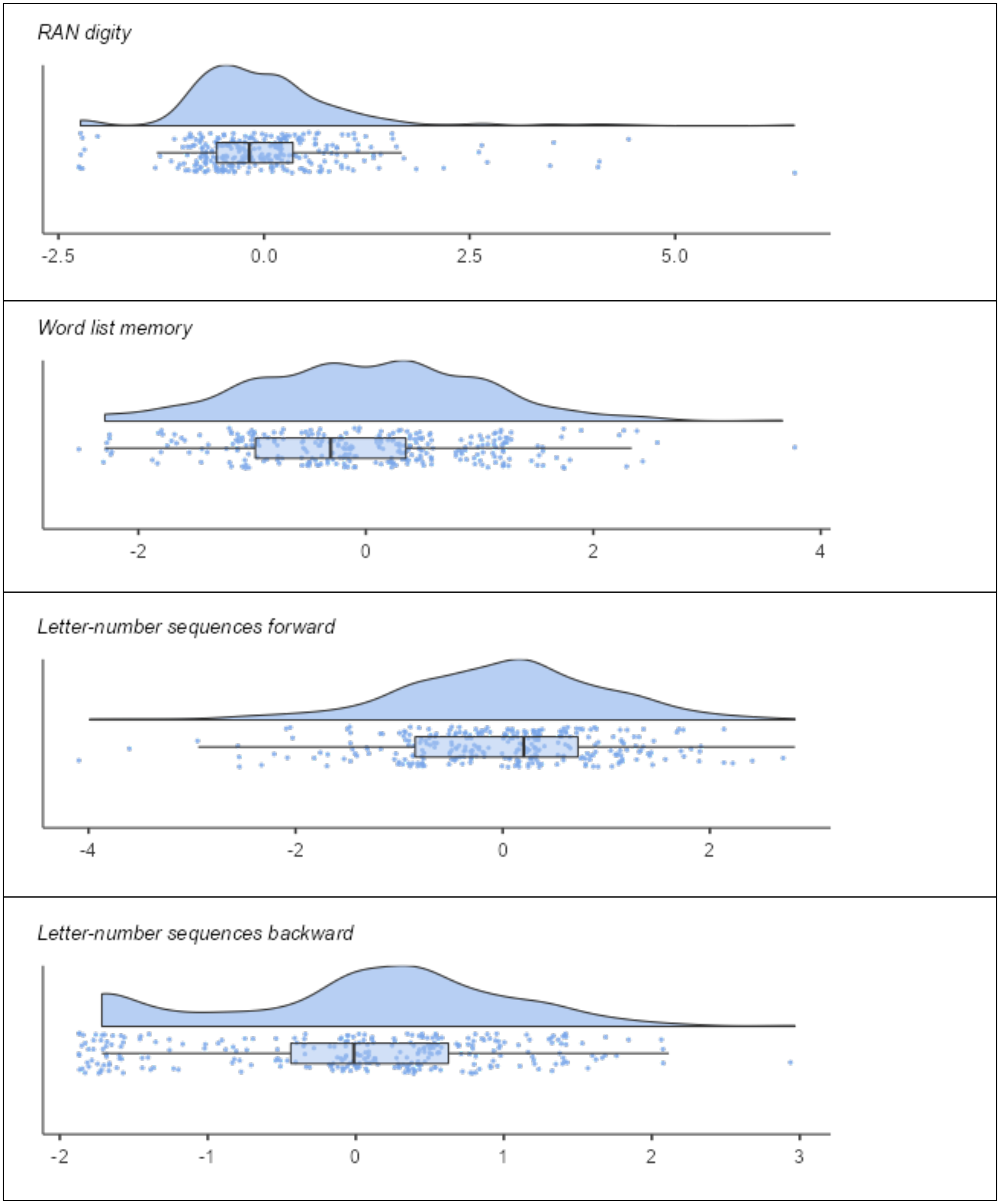
RAN digity
-2.5
0.0
2.5
5.0
Word list memory
-2
0
2
4
Letter-number sequences forward
-4
-2
0
2
Letter-number sequences backward
-2
-1
0
1
2
3

References

The jamovi project (2021). *jamovi*. (Version 2.2) [Computer Software]. Retrieved from https://www.jamovi.org.
